# Biomarkers’ Responses to Reductive Dechlorination Rates and Oxygen Stress in Bioaugmentation Culture KB-1^TM^

**DOI:** 10.3390/microorganisms6010013

**Published:** 2018-02-08

**Authors:** Gretchen L. W. Heavner, Cresten B. Mansfeldt, Garrett E. Debs, Sage T. Hellerstedt, Annette R. Rowe, Ruth E. Richardson

**Affiliations:** 1 School of Civil and Environmental Engineering, Cornell University, Ithaca, NY 14853, USA; gheavner@gmail.com (G.L.W.H.); mans0094@gmail.com (C.B.M.); garrett.debs@yale.edu (G.E.D.); sage.hellerstedt@gmail.com (S.T.H.); 2 Department of Microbiology, Cornell University, Ithaca, NY 14853, USA; a.knee.row@gmail.com

**Keywords:** organohalide respiration, Dehalococcoides, trichloroethene, biomarkers, Geobacter, proteomics

## Abstract

Using mRNA transcript levels for key functional enzymes as proxies for the organohalide respiration (OHR) rate, is a promising approach for monitoring bioremediation populations in situ at chlorinated solvent-contaminated field sites. However, to date, no correlations have been empirically derived for chlorinated solvent respiring, *Dehalococcoides mccartyi* (DMC) containing, bioaugmentation cultures. In the current study, genome-wide transcriptome and proteome data were first used to confirm the most highly expressed OHR-related enzymes in the bioaugmentation culture, KB-1^TM^, including several reductive dehalogenases (RDases) and a Ni-Fe hydrogenase, Hup. Different KB-1™ DMC strains could be resolved at the RNA and protein level through differences in the sequence of a common RDase (DET1545-like homologs) and differences in expression of their vinyl chloride-respiring RDases. The dominant strain expresses VcrA, whereas the minor strain utilizes BvcA. We then used quantitative reverse-transcriptase PCR (qRT-PCR) as a targeted approach for quantifying transcript copies in the KB-1^TM^ consortium operated under a range of TCE respiration rates in continuously-fed, pseudo-steady-state reactors. These candidate biomarkers from KB-1^TM^ demonstrated a variety of trends in terms of transcript abundance as a function of respiration rate over the range: 7.7 × 10^−12^ to 5.9 × 10^−10^ microelectron equivalents per cell per hour (μeeq/cell∙h). Power law trends were observed between the respiration rate and transcript abundance for the main DMC RDase (VcrA) and the hydrogenase HupL (R^2^ = 0.83 and 0.88, respectively), but not transcripts for 16S rRNA or three other RDases examined: TceA, BvcA or the RDase DET1545 homologs in KB1^TM^. Overall, HupL transcripts appear to be the most robust activity biomarker across multiple DMC strains and in mixed communities including DMC co-cultures such as KB1^TM^. The addition of oxygen induced cell stress that caused respiration rates to decline immediately (>95% decline within one hour). Although transcript levels did decline, they did so more slowly than the respiration rate observed (transcript decay rates between 0.02 and 0.03 per hour). Data from strain-specific probes on the pangenome array strains suggest that a minor DMC strain in KB-1™ that harbors a *bvcA* homolog preferentially recovered following oxygen stress relative to the dominant, *vcrA*-containing strain.

## 1. Introduction

Groundwater contaminated with perchloroethene (PCE) and trichloroethene (TCE), two of the most common subsurface contaminants globally, can be successfully treated using in situ bioremediation [1]. Microbial strains, including *Dehalococcoides mccartyi* strain 195 (DMC195), can reductively dechlorinate carcinogenic PCE to nontoxic ethene [2,3,4]. Other *Dehalococcoides mccartyi* (DMC) strains [5,6,7,8], including the multiple strains in the mixed culture KB-1™ [9], specialize in individual steps in the pathway [10,11]. Degradation of PCE or TCE often stalls at dichloroethene (DCE) or vinyl chloride (VC). For example, the VC to ethene step is cometabolic for both DMC195 and DMC strain FL2 [3,5]. However, VC is used for growth in other DMC strains, including BAV1, VS, and strains in KB-1™. Because VC is a primary contaminant of concern, bioaugmentation cultures such as the KB-1™ consortium have been widely used in chloroethene bioremediation to prevent accumulation of VC. However, ensuring effective remediation by these cultures in situ remains a challenge, and methods to estimate the in situ activity of the appropriate DMC strains (i.e., VC to ethene specialists) are needed.

To demonstrate sufficiently that bioremediation is occurring at a site, three criteria must be met: loss of contaminant mass from the site must be demonstrated, organisms that are capable of degrading the contaminant must be present, and the capability of the organisms to degrade the contaminant in situ must be exhibited [12]. The third piece of evidence is the most difficult to demonstrate conclusively. RNA and protein biomarkers may serve as the key piece of evidence and may even inform quantitative forecasting of in situ rates of bioremediation. A range of studies have examined reductive dehalogenase (RDase) gene expression in a lab setting [13,14,15,16,17,18,19,20,21,22], and select studies have also successfully detected RDase gene transcripts [14,23,24] or VC-oxidizing enzyme transcripts [25] in soils and sediments. Based on these studies, a suite of potential biomarkers has been generated and includes RDases and other oxidoreductase targets such as the NiFe hydrogenase, Hup [26]. RNA transcripts from genes linked to the VC-to-ethene step of reductive dechlorination (VC-RDases VcrA and BvcA) are particularly promising biomarkers for the complete remediation of chlorinated ethenes [26]. However, broader testing is needed especially since correlations between VC dechlorination and gene expression activity have yet to be investigated. Such trends would be highly valuable for monitoring complete in situ bioremediation of chlorinated ethenes.

The correlation between respiration rate and mRNA transcript number has been extensively studied for DMC195 in the Cornell mixed culture (D2) [13,16,22]. However, because of the variation in substrates and RDases between DMC strains, finding a single robust biomarker for reductive dechlorination of chloroethenes is challenging. In this study, we have expanded previous work with DMC195 [13,16,22] to include similar mRNA transcript abundance studies with the *Dehalococcoides*-containing mixed culture KB-1^TM^ that is used commercially for bioaugmentation at field sites. KB-1^TM^ differs from the D2 culture in that it contains multiple DMC strains in addition to an organohalide respiring species of *Geobacter.* Furthermore, distinct RDases are transcribed when the KB-1 lab cultures maintained at the University of Toronto (KB1-UT) are fed TCE, *cis-*DCE, VC, or 1,2-dichloroethane [11,17,27]. Notably, enzymatic activity of BvcA using native enzyme gels from KB-1^TM^ enrichment cultures was only detected in enrichment cultures maintained on *cis*-DCE, whereas VcrA activity was detected in all enrichment cultures including the culture maintained solely on VC [11]. All transcriptomic and proteomic studies in DMC thus far have also found high expression of the Ni-Fe hydrogenase, Hup, and current theories suggest direct involvement of Hup in the electron transport chain [28,29]. All DMC studied to date use only H_2_ as the electron donor and acetate as the carbon source. However many cultures are fed organic substrates (e.g., ethanol, butyrate, lactate) which can be fermented by other organisms in the mixed cultures to produce H_2_.

The primary goal of this work is to test the robustness of selected RNA biomarkers as indicators of actual chlorinated ethene organohalide respiration rate in DMC strains. In this work we first utilized high throughput microarray and shotgun proteomics methods to characterize the KB-1 expression profiles and help select final RNA targets for RT-qPCR assays in continuously fed cultures in which the respiration rate and mRNA levels were both in pseudo-steady-state. Understanding the decay rates of RNA biomarkers is important for interpreting RNA as a sensitive, dynamic biomarker at field sites in which environmental conditions may vary over weeks or months. Therefore we additionally examined the decay of the RNA biomarker signal following oxygen stress and during the eventual reactivation of a subset of DMC strains in KB-1.

## 2. Materials and Methods

### 2.1. Analytical Methods

Chlorinated ethenes, ethane, and methane were analyzed from 100-μL headspace samples via gas chromatography as described previously [15,22]. Hydrogen was analyzed from 50-μL headspace samples via reduction gas detector (RGD, Trace Analytical RGD2, Menlo Park, CA, USA) or by a thermal conductivity detector (PerkinElmer, San Jose, CA, USA) at higher concentrations [30,31]. During the oxygen stress experiment, oxygen was analyzed via gas chromatography as described by Gossett [32].

### 2.2. Microbial Culture

KB-1^TM^ culture was provided by SiREM Labs of Guelph, Ontario, Canada. The culture contains multiple DMC strains, a *Geobacter* strain, and a wide variety of other microorganisms [33,34,35]. A metagenome library is available for a related lab culture at the University of Toronto [36] (DCKB1, http://genome.jgi-psf.org/aqukb/aqukb.home.html).

### 2.3. Continuous Feed Experiments

Continuous TCE feed experiments were conducted as 100 mL subcultures of undiluted KB-1^TM^ culture with 60 mL of anaerobic headspace in 160 mL, anaerobic serum bottles as described previously [22,37]. Parameters for the experiments are presented in Table S1. Methanol and ethanol (4 subcultures) or hydrogen (6 subcultures) served as electron donors and were continuously fed in excess of stoichiometric needs for TCE dehalogenation to ethene at a minimum molar ratio of 4 to 1.

### 2.4. Oxygen Stress Experiment

Six 100 mL subcultures were batch-fed electron acceptor (2 µL neat TCE). Electron donor (H_2_), and a carbon source, acetate, were fed in excess. After one full dechlorination cycle to ethene (approximately 24 h), one control bottle was sacrificed for proteomic and microarray analysis. The remaining cultures were purged anaerobically, re-fed, and, in the four experimental bottles, 2.4 mg air was added which corresponded to 1.6 mg/L dissolved oxygen at equilibrium. The second, unstressed control was sacrificed after the second dose of TCE had been converted to a mix of *cis*-DCE, VC, and ethene (approximately 50 h). Two experimental bottles were sacrificed for proteomic and microarray analysis approximately eight hours after the stressor was added (before any of the second TCE dose was transformed). The remaining two bottles were sacrificed approximately 170 h after stress, when *cis-*DCE was the dominant compound and VC and ethene were starting to be produced (suggesting that the DMC populations were recovering). Parameters for the oxygen stress experiments are presented in Table S1.

### 2.5. Calculation of Respiration Rates

Respiration rates in terms of electron equivalents (eeq) were based on measured chloroethenes and utilized the following formula for TCE:

(1) $$rTCE=\frac{\Delta (2*DCE+4*VC+6*ETH)}{\Delta t}$$

where *r*TCE is the TCE respiration rate (μeeq/L∙h), Δ*t* is the timestep length, and DCE, VC, and ETH are the *cis*-DCE, vinyl chloride, and ethene concentrations (μmol/L), respectively. Normalization to cell density was done using qPCR-based DNA quantities of DMC cells.

### 2.6. Nucleic Acid Extraction and Quantification

DNA and RNA extractions were performed using the AllPrep DNA/RNA Mini Kit (Qiagen, Hilden, Germany) with modifications and DNase treatments as previously described [15,22]. Total DNA was quantified using the Quant-iT^TM^ Picogreen^®^ double stranded DNA assay (Invitrogen, Waltham, MA, USA). RNA quality and quantity was analyzed using the Agilent 2100 BioAnlyzer (Agilent, Santa Clara, CA, USA). DNase treatment, cDNA synthesis, qPCR set up, and qPCR run conditions were performed as previously described [13,16,22]. Prior to qPCR, all DNA and cDNA samples were diluted 1 to 5 with molecular grade water. Primers and annealing temperatures used in this study are listed in Table S2. Primers to amplify all known orthologs of *hupL* and DET1545 were designed using tools from Integrated DNA Technologies (IDT) and gene sequence alignments of diverse orthologs. Luciferase mRNA was spiked into samples and used to estimate overall mRNA recovery and reverse transcription efficiency [21]. Transcript levels were calculated from raw fluorescence data using the Data Analysis for Real-time PCR (DART-PCR) method [16,38,39]. Long amplicons (for 16S, *tceA*, DET1545 homologs, *vcrA*, *bvcA* and degenerate *hupL* primers) or D2 mixed culture DNA extracts (for DMC195-targetting *hupL* primers) were used to generate standard curves for each target. Primers and annealing temperatures used to generate long amplicons are listed in Table S3.

### 2.7. Microarray Analysis

Microarray analysis including preparation of cDNA, hybridization conditions, and data normalization were performed as previously published [37,40]. The data for this study is freely available at the NCBI Gene Expression Omnibus under the experimental series GSE42136 for the experiments GSM1033361-66. The pangenome array used [41] contained multiple probes for orthologous genes, and BLASTn analyses was used to determine the strain specificity of the various specific probes.

### 2.8. Proteomic Analysis

Protein extractions were performed on cell pellets of 50 mL culture (14,000× *g*, 10 min). The supernatant was decanted and the pellet was frozen at −20 °C, and shipped overnight on dry ice to the Environmental Molecular Sciences Laboratory (EMSL) at the Pacific Northwest National Laboratory for proteome extraction and analysis. Details of the sample digestion, Isobaric Tag peptide labeling, and 2D-LC-MS/MS analyses are provided in the Supplemental Methods. MS/MS data were searched using SEQUEST software [42] against a peptide database constructed from a series of DMC isolate genomes and metagenomic datasets and other known RDase sequences, using relatively conservative filters [Xcorr values of 1.9 (+1), 2.2 (+2) and 3.5 (+3)]. Resulting peptide identifications were filtered using an MS-GF cutoff value of 1 × 10^−10^ [43]. For the oxygen stress experiment data, Tandem Mass Tag (TMT) reporter ion intensities, acquired using the software tool MASIC (MS/MS Automated Selected Ion Chromatogram [44]), were used to measure relative peptide abundance across samples [45]. Aggregation of the relative abundance measurements for all peptide spectra assigned to a given protein was used to measure the relative amounts of each of the identified proteins. Relative protein quantities of biomarkers in shotgun proteomic analyses (both those with and without TMT tags) were estimated by calculating the normalized spectral abundance factor (NSAF) and/or by parent ion intensity [46].

## 3. Results and Discussion

### 3.1. Proteomic and Transcriptomic Profiling of KB-1^TM^

We first examined proteomic and transcriptomic datasets to help prioritize targets for the examination of trends between respiration rate and mRNA transcript levels (as determined by RT-qPCR). Baseline gene expression in the KB1^TM^ DMC populations was determined by employing DMC-pangenome microarrays [41]. Additionally, we monitored protein levels for multiple organohalide respiring community members via shotgun metaproteomic surveys compared against available metagenomes (KB1^TM^), pure culture genomes of DMC, and RDase databases. Metaproteomic data demonstrate expression of diverse DMC RDases in KB1^TM^ although at widely varying concentrations (NSAF scores are presented in Table 1). Based on NSAF values, the most abundant DMC protein overall in KB-1^TM^ was the VC-dehalogenase VcrA (NSAFs of 4.72 × 10^−2^). Other abundant RDases were DET1545 homologs followed by a different VC-dehalogenase, BvcA (NSAFs of 4.68 × 10^−2^ and 9.23 × 10^−3^, respectively). Twelve other RDase homologs were also found in the metaproteome at lower abundance, including the *Geobacter* PCE-dehalogenase, PceA (Table 1). The TCE-dehalogenase, TceA, was detected, but at a low abundance (NSAF of 4.88 × 10^−4^), notably when compared to previously analyzed samples from the DMC195-containing D2 culture (NSAF of 0.152) [22]. This is consistent with recent studies showing that the *tceA* gene is on a minor DMC strain’s genome in KB1^TM^ [11]. The HupL homologs were the third most abundant biomarker of interest detected (NSAF = 9.59 × 10^−3^). Additionally there was strong evidence for expression of the formate-dehydrogenase like (“Fdh”-like) oxidoreductase (NSAF = 4.62 × 10^−3^ for the alpha subunit). Although the exact function of “Fdh” in DMC remains unknown, this oxidoreductase is proposed to be involved in enzyme complexes critical to DMC’s electron transport chain [29,37]. With respect to the high expression levels of respiratory enzymes in DMC these protein abundance findings agreed with previously published omics datasets [28,47]. For perspective, the corresponding values for two housekeeping genes, the chaperonin GroEL and the translation elongation factor Tuf, were NSAF values of 8.10 × 10^−3^ and 5.97 × 10^−3^, and microarray intensities of 44,596 and 23,149, respectively.

**Figure 1:**
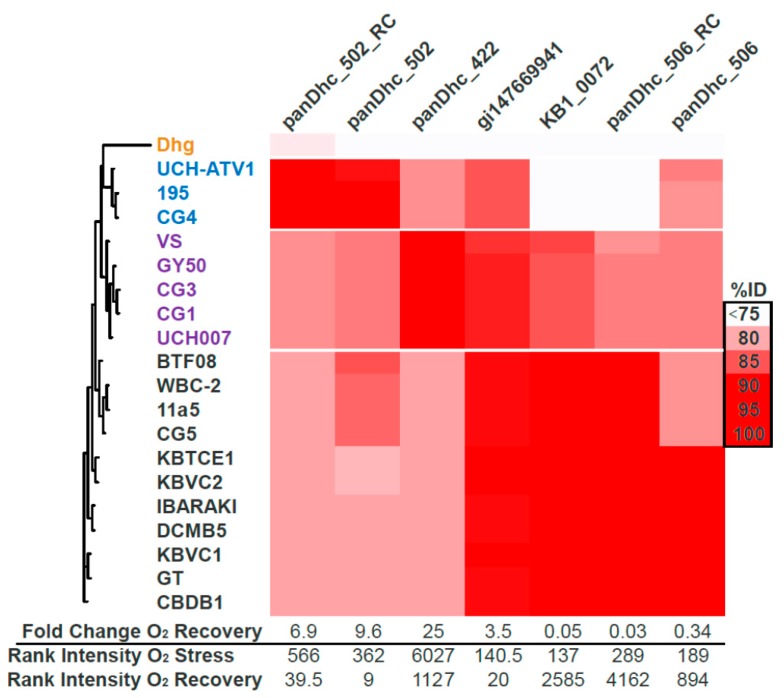
Heatmap coloring the percent identity of the microarray probes targeting the DET1545 RDase homologs aligned to full length sequences of DET1545 homologs encoded on full genomes available for *DMC*. The DET1545 homologs are ordered according to a CLUSTAL OMEGA alignment of the full length sequences with default parameters, and the phylogenetic tree is presented. The text color represents the groupings within *DMC*: Cornell (blue), Victoria (purple), Pinellas (black), and a *Dehalogenimonas* outgroup (orange). The fold change following recovery from the oxygen stress and the rank of the microarray probe intensity at the onset and after the recovery from the oxygen stress is presented in the table below the heatmap.

**Figure 2:**
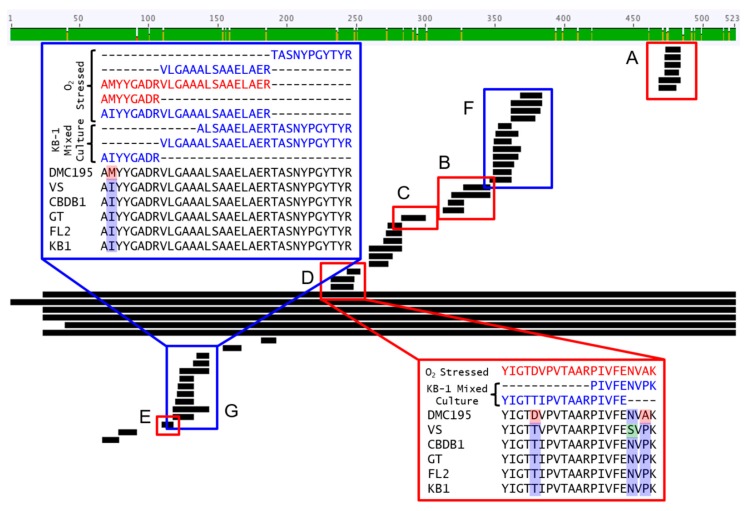
A comparison of RDase DET1545-homolog peptides (short black bars) detected in the KB-1 culture sample and oxygen stressed KB-1 culture to the full length 1545-homolog sequences in DMC genomes in different groups: 1545-Cornell (DMC195), 1545-Victoria (DMC strain VS), and 1545-Pinellas (DMC strains CBDB1, GT, FL2, and the KB-1 mixed culture). Peptides boxed in red (**A**–**E**) show strain specificity, whereas peptides boxed in blue (**F**,**G**) are highly conserved and align across all strains. In the insets, amino acid residues where variation occurs are highlighted in red if specific for DMC195, green if specific for VS, or blue if not strain-specific. This figure was created using Geneious version 5.0.4 created by Biomatters.

Despite their unknown substrate range, DET1545-like dehalogenases are notable because of the enzyme’s clear expression in KB-1^TM^ (“rdhA5” in KB-1 [11]), homologous RDases (e.g., cbdbA1638 and DhcVS_1436) are encoded on the genomes of a majority of DMC strains sequenced, and the 1545 phylogeny maps well to the genome level subspeciation (Figure 1). These considerations are in contrast to the narrow strain distribution of most RDases of known function and importance for chloroethene bioremediation (e.g., TceA, VcrA, BvcA). Additionally, DET1545 homologs and RNA transcripts have previously been detected at field sites [14]. In the metaproteome of the KB-1^TM^ culture, 46 DET1545 homolog peptides’ spectra were detected. Data confirm that multiple DMC strains in KB-1^TM^ are expressing distinct 1545-like orthologs at the protein level (RNA trends also supported this conclusion). When we align detected peptides with homologs from the Cornell, Victoria, and Pinellas subgroups of DMC (1545-C, 1545-V, and 1545-P, respectively), some peptides display phylogenetically informative signals whereas others were in regions too conserved to resolve strains (Figure 2). Twenty-five of the detected peptides align with all homologs (Regions F & G in Figure 2), but others are not as conserved and are strain or cluster specific (Regions A to E in Figure 2). A minor DMC strain in KB-1^TM^ could be differentiated by virtue of slight differences in the DET1545 homologs’ peptides (Table S4 and Figure 2). In region A, six peptides were detected: one was specific for 1545-C and the other five were specific for 1545-P including the homolog on the *vcrA*-harboring dominant DMC strain in the metagenome of the KB1-UT mixed culture. In other variable regions B to E, only the Pinellas-type peptides were confirmed, although the minor variants may still be present. In summary of these data, at least two variants of the DET1545 homolog are expressed in KB-1™. One dominant variant is Pinellas-like, and the other minor one is Cornell-like. Similarly, Hup peptide and transcript variants were likewise observed [48]. The omics results informed selection of targets and design of primers for qPCR in subsequent studies (including new degenerate primers for capturing diverse *hupL* sequences).

### 3.2. Correlations Between mRNA Biomarkers and Respiration Rate

**Figure 3:**
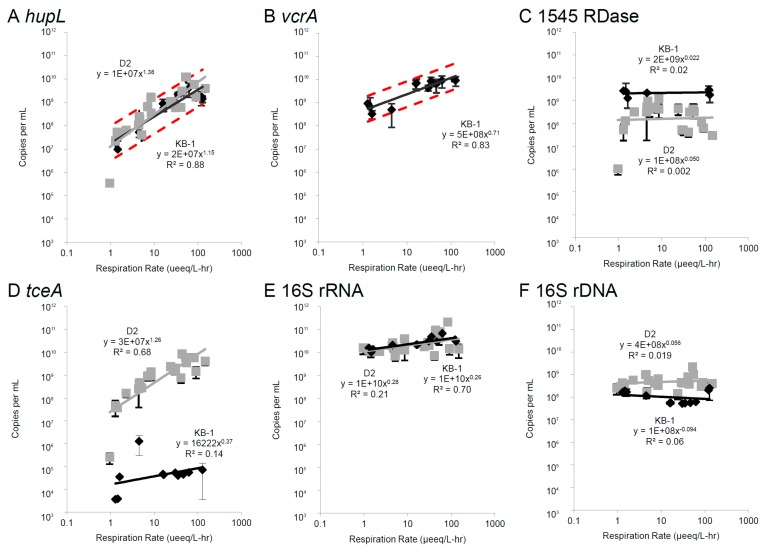
Pseudo-steady-state mRNA concentrations (copies per mL) of specific targets: hydrogenase *hupL* (**A**); reductive dehalogenases *vcrA* (**B**), 1545 (**C**), and *tceA* (**D**); and 16S rRNA units (**E**) and 16S rRNA genes (**F**) vs. steady-state respiration rates (μeeq/L∙h) for the KB-1 culture (black diamonds) and D2 culture (grey squares). Error bars represent standard deviations of PSS mRNA measurements over time (Y-error bars). Power law trend lines for KB-1 (solid black line) and D2 (solid grey line) and 95% confidence intervals around the KB-1 trend (dashed red line) are displayed where appropriate. Note that reductive dehalogenase *vcrA* is not present in the D2 DMC strain’s genome. Corresponding trends with *bvcA* and *hupL* degenerate primers are shown in Figure S2.

Targeted quantitative approaches (RT-qPCR) were used to examine relationships between mRNA expression levels and actual culture respiration rates for TCE. Subcultures of KB-1^TM^ were continuously fed electron acceptor (TCE) with excess electron donor (methanol, ethanol, hydrogen) and carbon source (Table S1). Over the course of these approximately 24 h experiments, RNA biomarkers first increased then reached a pseudo-steady-state (PSS) concentration as in previous studies with culture D2 [16]. DMC cell density in KB-1 (as measured by 16S rRNA gene copies per mL) remained nearly constant at ~1 × 10^8^ copies/mL (Figure 3F). Although respiration rates ranged from 1.6 to 128 µeeq/L∙h (9.00 × 10^−13^ to 2.88 × 10^−12^ µmol/cell∙h), steady-state ribosome content (per mL) also remained relatively constant (equal to approximately 100 copies per cell) and was indistinguishable from the D2 culture data (Figure 3E). Cell densities (based on 16S rRNA gene copies per mL) did not change significantly within or across experiments and were slightly lower than in the D2 mixed culture [49] (Figure 3F). This constant value indicates that the changes in the mRNA levels are because of up-regulation and can be related to the respiration rates, not an increase in cell density. RNA half-lives estimated for transcripts in D2 [22] and KB-1 (see section below on mRNA decay) suggest turnover times of 10–30 h for mRNA.

Although no significant correlations are seen for *bvcA*, *tceA* or 1545∙homolog transcripts, trends were observed in KB1^TM^ for *hupL* and *vcrA* with correlation scores (Pearson’s R^2^) of 0.88 and 0.83, respectively, across two orders of magnitude in respiration rate (Figure 3), suggesting these are the most promising of the tested RNA biomarkers for respiration rate in KB-1. Significant correlations were previously observed for *hupL* and *tceA* mRNA biomarkers in the DMC195 containing D2 culture and are shown in Figure 3 for comparison (note that *vcrA* and *bvcA* are not found in the DMC195 genome or D2 metagenome) [13,16,22]. *hupL* expression trends with respiration rate in the KB-1^TM^ culture was indistinguishable from trends in the D2 culture over more than two orders of magnitude of respiration rate and corresponded to a range of 0.1 to 10 mRNA copies per cell. These trends existed regardless of whether cDNA pools were amplified with previously described qPCR *hupL* primers (perfect matches only to DMC195 [16]; Figure 3A) or our newly designed degenerate *hupL* primers that amplify diverse DMC *hupL* sequences (Table S2 and Figure S1A). Because of the importance of molecular hydrogen (H_2_) in anaerobic microbial ecology in general and organohalide respiring communities in particular, researchers have designed microarrays to examine hydrogenase transcript levels in different hydrogenotrophs in mixed communities [50]. The current work suggests that *hupL* transcript levels may serve as a robust quantitative biomarker of activity across many DMC strains.

Turning from the hydrogenase to dehalogenases, *vcrA* transcript levels ranged from 2 to 50 copies per cell. The DET1545 RDase homologs and *bvcA* were expressed at high and nearly constant levels (averaging 30 and 60 copies per cell, respectively) regardless of respiration rate (Figure 3 & Supplemental Figure S1). It is interesting to note that the protein levels for these RDases (Table 1) were well below those for VcrA despite high constitutive expression at the RNA level. Translation rate differences across RDase transcripts has been seen in DMC before with TceA showing higher translation than other DMC 195 RDase transcripts [22]. For the DET1545 homolog in KB-1™, the consistent expression level across respiration rates is in sharp contrast to the expression pattern observed for the D2 culture, in which the DMC195 DET1545 homolog expression peaks at a low to mid respiration rate (Figure 3C) [13,22]. The *tceA* gene was expressed in KB-1^TM^ at a low level (less than 0.01 transcripts per cell), notably when compared to the D2 culture (Figure 3D). The current work demonstrates the first quantitative correlation between transcripts of a VC RDase (in this case *vcrA*) and pseudo-steady-state organohalide respiration rate.

The *Geobacter* RDase *pceA* was not examined by qPCR in this study. *Geobacter* is a non-*Dehalococcoides* dechlorinating population in the KB-1^TM^ culture that can dechlorinate PCE and TCE to *cis*-DCE via a PceA enzyme [35]. Since chloroethene bioremediation is most concerned with the VC-to-ethene step of the dechlorination process and *Geobacter* does not dechlorinate beyond *cis*-DCE, *Geobacter*’s RDase was not explored as a biomarker in these RNA studies. However, the proteomic evidence is strong that PceA is abundant in the KB1 culture—consistent with other recent reports [11]. This result may suggest that *Geobacter* is primarily responsible for degradation of PCE to DCE and thus explain why no trend with respiration rate was seen in KB-1^TM^ for the TCE-to-cDCE dehalogenase of DMC strains, TceA.

### 3.3. Oxygen Stress Response

**Figure 4:**
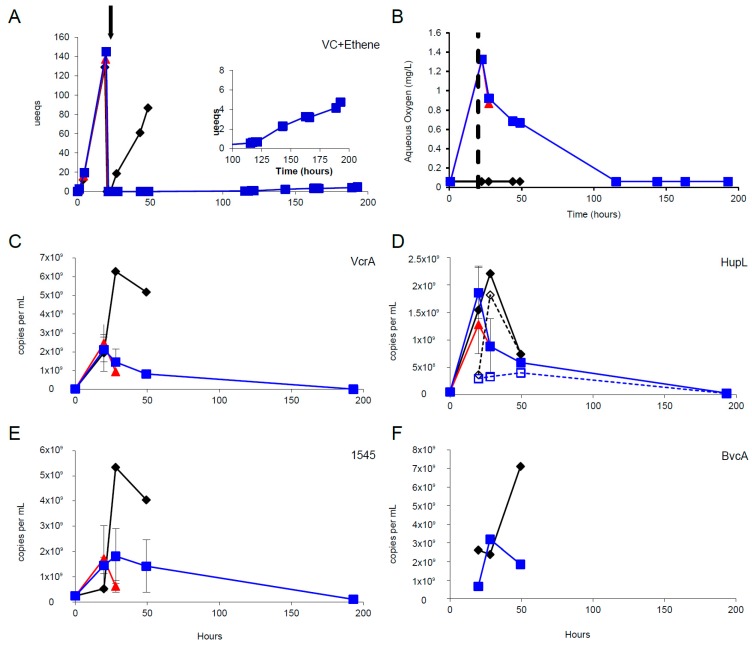
Timecourses of metabolite (**A**), oxygen (**B**), and mRNA (**C**–**F**) concentrations for specific targets in oxygen stress experiments. The arrow in A (vertical dashed line in B) indicates when the stress was added at 20 h. The controls are in black diamonds, and the stressed bottles are in red triangles (sacrificed ~12 h after stress) and blue squares (sacrificed at end of the experiment). Note that VC + ethene production is recovering at ~100 h after the oxygen stress (inset in panel A). Error bars represent standard error of duplicate reactors. For *hupL* in panel D, results from degenerate primer sets (dashed lines) are plotted on the same set of axes. Full dechlorination profiles are shown in Figure S3.

We examined how mRNA biomarkers decay in DMC when cells experience severe stress. Decay rates for RNA help test the assumption that RNA pools had reached pseudo-steady-state levels in the continuous feed experiments. Additionally they will help in interpreting persistence of signal in field settings that could lead to overestimation of actual current in situ respiration rates. To examine how rapidly mRNA biomarkers decay in KB1’s DMC populations when cells experience severe stress, active cultures were oxygen-stressed. The six subcultures in this experiment had batch respiration rates of 69.4 ± 5.6 μeeq/L∙h prior to the addition of oxygen (Figure 4A). After headspace purging and re-feeding, the control (without oxygen added) resumed rapid respiration (29.9 μeeq/L∙h), but bottles with oxygen added had no measurable dechlorination activity (Figure 4A and Figure S2). Oxygen levels for the six subcultures (Figure 4B) were in agreement with the color of the redox dye, resazurin, in the medium. The measured respiration rates for cultures sacrificed 8 h after addition of the stressor dropped substantially (0.39–0.44 μeeq/L∙h). The culture became anaerobic again at between 48 and 115 h, and organohalide respiration resumed. TCE degradation to *cis*-DCE occurred in this time period (Figure S2). However, this dechlorination step may have been performed exclusively by the PceA-expressing *Geobacter* strain present in the mixed culture which survived the oxygen stress. Ultimately, VC began to build up, suggesting that DMC strains were resuming respiration 170 h after the stressor was added (Figure 4A and Figure S2). The measured respiration rates for cultures sacrificed 170 h after the addition of the stressor were higher than the initial stressed cultures (2.2–2.5 μeeq/L∙h).

Time courses of target biomarker expression levels (*vcrA*, *hupL*, DET1545 homolog, and *bvcA*) are presented in Figure 4C–F. For all targets, the control bottle remained at a higher expression level longer than the experimental bottles sacrificed either 8 or 170 h after stress. The eventual decrease in transcript abundance for the control bottle is consistent with previous results showing starvation of the batch culture after chloroethenes were consumed [22]. The trend of *hupL* declining in transcripts more rapidly than RDases has also been seen previously [15].

### 3.4. mRNA Decay Under Oxygen Stress Conditions

RNA decay rates for DMC were calculated from batch reactors after oxygen addition inhibited the cultures. RNA decay rates in KB-1^TM^ for *vcrA*, *hupL*, and the DET1545 homolog (Figure S3) were found to be 0.02–0.03 per hour (half-lives of 20–30 h), similar to those observed previously for the DMC population in D2 following starvation (0.02–0.06 per hour) [22]. These RNA decay rates are much higher than cell DNA decay rates 0.003 to 0.004 per hour previously found for D2 [51].

### 3.5. Microarray Trends in the Oxygen Stress Experiment

Microarray analysis of the oxygen stress experiment showed that in the cultures that were sampled at 170 h (when DMC populations started recovering activity), specific RDases had higher expression relative to just after stress (bottles sacrificed at 12 h): homologs of DET0173, DET1545, and *bvcA*. The most downregulated RDases were the DET1545 homolog and *vcrA*. The DET1545 homolog being both highly up and down-regulated is again explained by the presence of multiple strains of DMC in the KB-1^TM^ and KB1-UT cultures [11,17,52,53]. The DET1545 homolog probes showing the strongest increase in intensity target 1545-C and 1545-V type sequences, whereas those decreasing the most target 1545-Pinellas variants (Figure 1), suggesting that the P-type DMC strain fails to recover while at least two minor strains (one Cornell and one Victoria) start to take over. Other studies have shown shifts in DMC strain ecology following starvation and oxidative stress from permanganate [54,55].

### 3.6. Multiplexed Proteomic Analysis of Oxygen Stress Experiments

**Figure 5:**
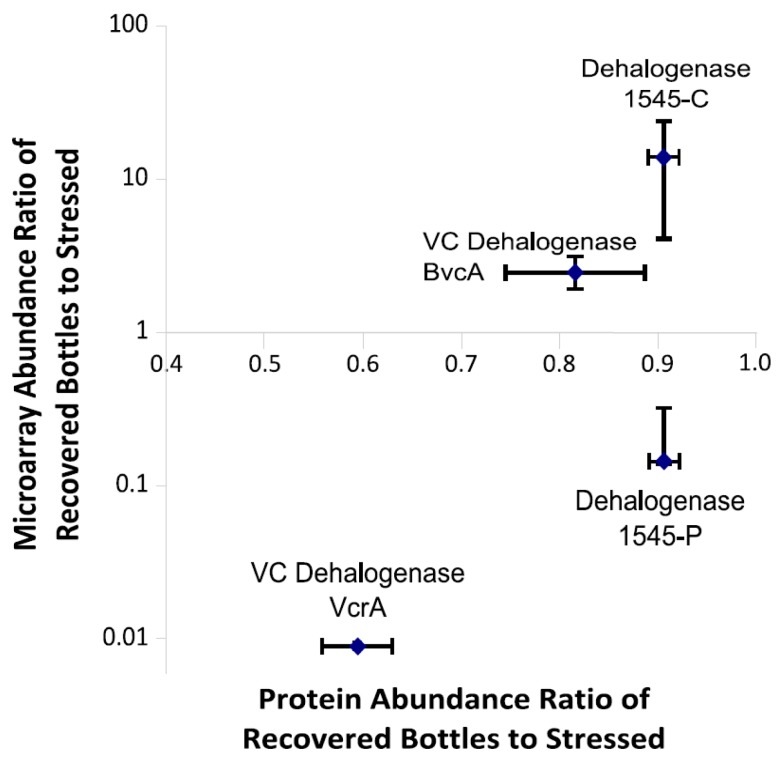
Microarray probe intensity ratio of recovered bottles to stressed bottles vs. protein abundance ratio of recovered bottles to oxygen stressed bottles for the reductive dehalogenases detected in both the protein and microarray results (1545, BvcA and VcrA only). 1545-C and 1545-P refer to microarray probes specific for the Cornell and Pinellas clusters, respectively.

Similarities were observed between the microarray results and multiplex protein results for the oxygen stress experiment. The proteomes of recovering cultures (170 h) were compared to those from 8 h post-stress. In both the microarray and protein datasets, the biomarker abundance ratio of the recovering:stressed cultures was high for BvcA and 1545-C/V probes of the DET1545 homolog and low for VcrA and the 1545-P specific probes (Figure 1 and Figure 5). These RDases were the only DMC RDases detected in both the proteome and transcriptome of the oxygen stressed bottles. Additionally, *Geobacter’s* PceA enzyme was detected in the proteome, but no probe for its mRNA was contained on the microarray. The stressed cultures were sacrificed only 8 h after the stress was added, which was likely not enough time for protein turnover to lower protein levels in samples. Protein decay rates were previously calculated for DMC RDases in the D2 culture, and were found to be approximately 0.0025 per hour—similar to DNA decay rates [22]. Thus, the cultures sacrificed at 8 h can be thought of as representative of the maximum protein levels observed. In the cultures sacrificed 170 h after the stressor was added, VcrA levels decreased dramatically, whereas the DET1545 homologs and BvcA were at similar levels to the cultures sacrificed at 8 h. As the DMC strains were inactive for a significant portion of this time prior to oxygen depletion, one would expect a decrease in enzyme levels because of decay. That is not the case for the peptides from DET1545 homologs and BvcA in the cultures at the end of the experiment, which are at similar levels to their maximum for this experiment. Thus, DMC strains with 1545-C/V and BvcA homologs appear to recover faster than the strain containing the VcrA and 1545-P type homolog. Diverse DMC strains are a commonly observed feature of organohalide respiring communities [53] and likely contribute to the robustness of the cultures as bioaugmentation agents.

### 3.7. Summary

In this study we used metaproteomic and microarray data to confirm the most highly expressed enzymes proposed to be involved in organohalide respiration of TCE by KB-1 and then examined transcript abundances of these putative biomarkers in continuously fed subcultures exhibiting a wide range of pseudo-steady-state respiration rates. We have established the first correlation between mRNA expression levels of a VC-RDase, in this case *vcrA* but not *bvcA*, and pseudo-steady-state respiration rate in any DMC culture. Additionally, using degenerate primers for a subunit of the DMC-wide hydrogenase, *hupL*, we found very similar power law correlation equations for multiple strains of DMC (KB-1^TM^ and D2). This finding is important because the diversity of unique RDases complicates their ability to be deployed as a universal biomarker of organohalide respiration when monitoring mixed communities or natural systems. Although the HupL hydrogenase does not directly dehalogenate the chlorinated compounds, it is involved in the oxidation of molecular hydrogen—the only known electron donor for DMC strains to date. It is proposed to be involved in the electron transport chain of DMC [29].

Knowing mRNA transcript decay rates in vivo helps predict persistence of signal upon perturbations that alter actual respiration rates. In this study, mRNA decayed directly after oxygen stress with rates between 0.02 and 0.03/h (half-lives of 20–30 h), suggesting that mRNA signals might decay over the order of days following severe stress. Furthermore, both transcriptomic and proteomic data indicated that after anaerobic conditions were reestablished, a DMC strain that harbors *bvcA* and a Cornell-group or Victoria-group DET1545 RDase homolog recovers while the originally-dominant strain with *vcrA* and a Pinellas-type DET1545 homolog does not. DMC strain diversity may improve the robustness of this and other bioaugmentation cultures against in situ stress at field sites.

## Figures and Tables

**Table 1:** Organohalide respiration biomarkers and selected housekeeping gene biomarkers detected by shotgun proteomics in a KB-1^TM^ metaproteome sample and corresponding pangenome microarray probe intensities. Protein intensity proxy is NSAF = Normalized Spectral Abundance Factor (times 10^5^) for all detected protein homologs. For pangenome microarray data the corresponding probe with the highest intensity is shown. With the exception of the hydrogenase, HupL, all listed biomarkers are reductive dehalogenases. *Geobacter*’s PceA gene was not on the pangenome microarray.

Homolog Name	Protein Intensity	Microarray Intensity
VcrA	4723	106,022
DET1545	4684	32,998
HupL, NiFe Hydrogenase	959	118,339
BvcA	923	153,080
Geobacter PceA	94	-
KB1_8 (KB1_1468), KB1_9 (KB1_0060)	66	801
DET0180	62	1212
KB1_1, KB13109_4 (KB1_0054)	57	2279
TceA	49	1298
KB13241_3 (KB1_1549)	13	76
DET1528	8.4	182
KB13241_7 (KB1_1589), KB1_7	7.7	845
DET1519	2.2	3635
DET1538	1.1	5536
cbdbA80	1.1	252

## Supplementary Materials

The following are available online at http://www.mdpi.com/2076-2607/6/1/13/s1.

Supplementary File 1
