# Supplementary material for: Biomarkers’ Responses to Reductive Dechlorination Rates and Oxygen Stress in Bioaugmentation Culture KB-1TM
Source: Microorganisms. 2018 Feb 8;6(1):13. doi: 10.3390/microorganisms6010013 (PMC5874627; doi:10.3390/microorganisms6010013)
Supplement: Supplementary File 1 [file microorganisms-06-00013-s001.pdf]

Supporting Information For:

Biomarkers' Responses to Reductive Dechlorination Rates and Oxygen Stress in Bioaugmentation Culture KB-1™

Gretchen L.W. Heavner, Cresten B. Mansfeldt, Garrett E. Debs, Sage T. Hellerstedt, Annette R. Rowe, Ruth E. Richardson

Supplementary Proteomics Methods

Urea Sample Digestion.

Cell pellets were resuspended in 9 M urea and vortexed into suspension. For cell lysis each sample was added to a barocycle pulse tube (Pressure Biosciences Inc., South Easton, MA) and barocycled for 10 cycles (20 seconds at 35,000 psi and then back to ambient pressure for 10 seconds). After transferring the supernatant to a centrifuge tube and spinning at 5,000 x *g* for 5 minutes to collect debris, the supernatant was transferred to a fresh tube and assayed with bicinchoninic acid (BCA) (Thermo Scientific, Rockford, IL) to determine the protein concentration. After reducing with 10 mM dithiothreitol (DTT) (Sigma, St. Louis, MO) at 60˚C for 30 minutes with constant shaking at 800 rpm, samples were then diluted 10-fold for preparation for digestion with 100 mM NH_4HCO_3; 1 mM $CaCl_2$ and sequencing-grade modified porcine trypsin (Promega, Madison, WI) were added at a 1:50 (w/w) trypsin-to-protein ratio for 3 h at 37˚C. The resulting peptides were cleaned using Discovery C18 (50 mg, 1 mL) solid phase extraction tubes (Supelco, St.Louis, MO) through the following protocol: 3-mL methanol was added for conditioning followed by 2-mL 0.1% TFA in H_2O. The samples were then loaded onto each column followed by 4 mL of 95:5: H_2O:ACN, 0.1% TFA, eluted with 1-mL 80:20 ACN:H_2O, and were concentrated down to ~30 µL using a Speed Vac. A final assay was performed to determine the peptide concentration. An equal mass

of each sample was aliquoted into fresh centrifuge tubes and were either directly analyzed by 2D-LC-MS/MS or were first labeled with TMT isobaric tags for comparative proteomics.

TMT Isobaric Tag Labeling.

The continuous-feed and stress experiments (sample sets of 6) were labeled using amine-reactive Thermo Scientific Tandem Mass Tag (TMT) Isobaric Mass Tagging Kits (Thermo Scientific, Rockford, IL) according to the manufacturer's instructions.[31] Tagged samples within each set were then combined and dried in the Speed Vac to remove the organic solvents, cleaned using Discovery C18 (50 mg, 1 mL) solid phase extraction tubes as described above, and once again BCA-assayed to determine the final peptide concentration.

2D-LC-MS/MS Analysis.

The 2D-LC system was custom built using two Agilent 1200 nanoflow pumps and one 1200 capillary pump (Agilent Technologies, Santa Clara, CA), Valco valves (Valco Instruments Co., Houston, TX), and a PAL autosampler (Leap Technologies, Carrboro, NC). Full automation was enabled by custom software that allows for parallel event coordination providing near 100% MS duty cycle through use of two trapping and analytical columns. All columns were manufactured in-house at EMSL by slurry packing media into fused silica (Polymicro Technologies Inc., Phoenix, AZ) using a 1 cm sol-gel frit for media retention. Mobile phases consisted of 0.1 mM NaH_2PO_4 (A) and 0.3 M NaH_2PO_4 (B) for the first dimension and 0.1% formic acid in water (A) and 0.1% formic acid in acetonitrile (B) for the second dimension.

MS analysis was performed using a LTQ Orbitrap Velos ETD mass spectrometer (Thermo Scientific, San Jose, CA) fitted with a custom electrospray ionization (ESI) interface with custom electrospray emitters.[32] The heated capillary temperature and spray voltage

were 275°C and 2.2 kV, respectively. Data were acquired for 100 minutes, beginning 65 minutes after sample injection and 15 minutes into gradient. Orbitrap spectra (AGC $1x10^6$) were collected from 400-2000 m/z at a resolution of 60k followed by data dependent ion trap CID MS/MS (collision energy 35%, AGC $3x10^4$) of the ten most abundant ions. A dynamic exclusion time of 60 seconds was used to discriminate against previously analyzed ions.

Data Analysis.

MS/MS data were searched using SEQUEST against a peptide database constructed from a series of DMC isolate genomes and metagenomic datasets and other known RDase sequences, using relatively conservative filters [Xcorr values of 1.9 (+1), 2.2 (+2) and 3.5 (+3)]. The following (meta)genomic databases were used in these searches: *Dehalococcoides mccartyi* strains CBDB1 and 195, the KB1-UT metagenomic sequences (DCKB1 at JGI's IMG website), D2 metagenomic sequences (PCEDH *and* PCEOT at JGI's IMG website), *Geobacter lovleyi*, *Methanoregula boonei*, *Methanosaeta thermophila*, *Methanospirillum hungatei*, *Spirochaeta thermophila*, *Sporomusa* str. KB1, *Syntrophomonas wolfei* and *Syntrophus aciditrophicus*. Resulting peptide identifications were filtered using an MS-GF[33] cutoff value of 1×10^{-10}.[33]

TMT reporter ion intensities, acquired using the tool MASIC (MS/MS Automated Selected Ion Chromatogram), were used to measure relative peptide abundance across samples.[31] The intensity associated with each reporter ion is proportional to the contribution of each of the component samples to the total peptide abundance. Aggregation of the relative abundance measurements for all peptide spectra assigned to a given protein was used to measure the relative amounts of each of the identified proteins.

Relative protein quantities of biomarkers in shotgun proteomic analyses (both those with and without TMT tags) were estimated by calculating the normalized spectral abundance factor (NSAF). This technique adjusts for biases in peptide detection arising from protein length and matrix effects in each MS/MS run.

Supporting Tables

Table S1. Psuedo-steady state and oxygen stress experimental parameters

Experiment	Culture Title	Length of Experiment (Hours)	EA	EA Feed Concentration (M)	Calculated EA Feed Rate (µeeq/L-hr)	ED	Carbon Source	Carbon Source Concentration (M)	Respiration Rate (µeeq/L-hr)	Respiration Rate After Stress (µeeq/L-hr)
KB1 2 Rates	K2A1	23.3	TCE	8.4	4.6	MeOH /EtOH	MeOH /EtOH	MeOH:48/EtOH:12	4.5	-
	K2A2	23.5			1.7				1.6	-
	K2C1	24.5			129				128.0	-
	K2C2	24.7			124				121.8	-
KB1 3 Rates	K3A1	26.7	TCE	1.2	16.4	H2	Acetate	37	16.0	-
	K3A2	27.0			16.6				16.5	-
	K3B1	27.2		3	42.8				35.3	-
	K3B2	27.4			39.3				30.5	-
	K3C1	27.6		8.4	99.9				46.7	-
	K3C2	27.8			98.2				62.5	-
Oxygen Stress Batch	O2A1	20.0	TCE	8.4	-	H2	Acetate	Neat	59.6	-
	O2A2	48.6			-				71.2	29.9
	O2B1	27.1			-				69.0	0.4
	O2B2	27.3			-				71.6	0.4
	O2C1	192.7			-				68.2	2.5
	O2C2	192.8			-				76.6	2.2

Table S2. mRNA biomarker targets with qPCR primer sequence and annealing temperature

Strain Targeted	Gene ID	Gene Name	Annotation	Primer Sequence (5'-3')	Annealing temp.	Amplicon length	Reference
All DMC strains	DET_DE16S	16S rRNA	16S ribosomal RNA	GGAGCGTGTGGTTTAATTCGATGC (sense) GCCCAAGATATAAAGGCCATGCTG (anti-sense)	60°C	270 bp	Fung, et al., 2007
Dehalococcoides mccartyi st. 195	DET0110	*hupL*	[Ni/Fe] hydrogenase, group 1, large subunit (EC:1.12.99.6)	TGACGTTATTGCAGTAGCTGAGT (sense) CACACCATAGCTGAGCAGGTT (anti-sense)	55°C	82 bp	Fung, et al., 2007
All DMC strains	DET0110	*hupL*	[Ni/Fe] hydrogenase, group 1, large subunit (EC:1.12.99.6)	TGACGTTATTGCAGTAGC(C/T)GA(A/G)(A/T) (sense) CACACCATA(A/G)CT(A/G)AGCAGGTT (anti-sense)	55°C	82 bp	This study
All DMC strains except MB	DET1545	1545	Reductive dehalogenase, putative	CGCTGCCGAACTGGCTGAAA (sense) GTTTTTACCGGAGCGGGGTC (anti-sense)	60°C	144 bp	This study
Dehalococcoides mccartyi st. 195 and FL2	DET0079	*tceA*	Reductive dehalogenase	TAATATATGCCGCCACGAATGG (sense) AATCGTATACCAAGGCCCGAGG (anti-sense)	60°C	317 bp	Fung, et al., 2007
DMC strains VS and GT *Dehalococcoides* containing mixed cultures KB-1 and ANAS	DCKB1_96900	*vcrA*	Reductive dehalogenase	GAAAGCTCAGCCGATGACTC (sense) TGGTTGAGGTAGGGTGAAAG (anti-sense)	60°C	205 bp	Waller, et al., 2005
DMC st. BAV1 and KB-1 Mixed Culture	BAV1_0847	*bvcA*	Reductive dehalogenase	AAAAGCACTTGGCTATCAAGGAC (sense) CCAAAAGCACCACCAGGTC (anti-sense)	60°C	92 bp	Ritalahti, et al., 2006

Table S3. Long amplicon targets for qPCR standards with primer sequence and annealing temperature

Organism	Gene ID	Gene Name	Annotation	Primer Sequence	Annealing temp.	Amplicon length	Reference
Dehalococcoides mccartyi st. 195	DET_DE16S	16S rRNA	16S ribosomal RNA	GATGAACGCTAGCGGCG (sense) GGTTGGCACATCGACTTCAA (anti-sense)	50°C	1377 bp	Hendrickson et al., 2002
All DMC strains	DET0110	*hupL*	[Ni/Fe] hydrogenase, group 1, large subunit (EC:1.12.99.6)	CGGATACTCCGCAACCTTATT (sense) (A/G)TCAGCCACAATCTTGCATTC (anti-sense)	55°C	942 bp	This study
All DMC strains except MB	DET1545	DET1545	Reductive dehalogenase, putative	TCAGCCGCGTCCCTGGTG (sense) GGCTTCACCCAGACCGGC (anti-sense)	50°C	823 bp	This study
Dehalococcoides mccartyi st. 195	DET0079	*tceA*	Reductive dehalogenase	ACGCCAAAGTGCGAAAAGC (sense) TAATCTATTCCATCCTTTCTC (anti-sense)	50°C	1732 bp	He et al., 2003
DMC strains VS and GT *Dehalococcoides* containing mixed cultures KB-1 and ANAS	DCKB1_96900	*vcrA*	Reductive dehalogenase	CTATGAAGGCCCTCCAGATGC (sense) GTAACAGCCCCAATATGCCAAGTA (anti-sense)	50°C	1482 bp	Muller et al., 2004
DMC st. BAV1 and KB-1 Mixed Culture	BAV1_0847	*bvcA*	Reductive dehalogenase	TGCCTCAAGTACAGGTGGT (sense) ATTGTGGAGGACCTACCT (anti-sense)	50°C	838 bp	Muller et al., 2004

Table S4. DET1545-ortholog peptides detected in KB-1^{TM} culture sample and the DMC strain homologs that they match. Spectral count is the number of spectra affiliated with the peptide during the shotgun metaproteome characterization. Peptides highlighted in green are specific for the Pinellas group, peptides highlighted in red are specific for the Cornell group. Peptides without highlighting match multiple groups.

Peptide No.	Peptide Sequence	Spectral Count in KB-1^{TM} Culture	Cornell Group DMC195	Victoria Group VS	Pinellas Group CBDB1, GT, KB-1 Mixed Culture	Pinellas Group FL2
13	IPLFNTYFYK	2			X	X
27	NIPLFNTYFYK	2			X	X
28	NVSLFNTYFYK	2	X			
26	NIPLFNTYFY	1			X	X
10	GTIANIPLFNTYFYK	6			X	X
9	GTIANIPLFNTY	3			X	X
14	LPLEPTHPIDAGIYR	1	X	X	X	X
21	LYGVLTDLPLEPTHPIDAGIYR	11	X	X	X	X
20	LYGVLTDLPLEPTHPIDAGIY	2	X	X	X	X
19	LYGVLTDLPLEPTHPID	1	X	X	X	X
37	TPEYGAPGR	2	X	X	X	X
36	TLTPEYGAPGRLYGVL	1	X	X	X	X
45	YTLTPEYGAPGR	2	X	X	X	X
25	LYTLTPEYGAPGRLYGVLTD	3	X	X	X	X
24	LYTLTPEYGAPGRLYGVL	1	X	X	X	X
23	LYTLTPEYGAPGRLY	1	X	X	X	X
22	LYTLTPEYGAPGR	1	X	X	X	X
30	QKLYTLTPEYGAPGR	5	X	X	X	X
46	YVGSEGGAAIMAGLGEASR	268	X	X	X	X
12	IGTIGNDARYVGSEGGAAIMAGLGEASR	5	X		X	X
31	QLIGTIGNDAR	1	X		X	X
44	YLGYQLIGTIGNDAR	136	X		X	X
32	SAGTLLGGMANGNTFYN	1			X	X
34	STQGSNELWR	1	X	X	X	X
15	LSTQGSNELWR	1	X	X	X	
11	IALSTQGSNELWR	1	X	X	X	
18	LVIPNVPLWEIALSTQGSNELWR	3	X	X	X	
17	LVIPNVPLWEIALSTQ	1	X	X	X	
16	LVIPNVPLWEIAL	1	X	X	X	
29	PIVFENVPK	1			X	X
43	YIGTTIPVTAARPIVFE	1			X	X
42	YIGTTIPVTAARPIVF	1			X	X
41	WTGTPEEASR	5	X		X	X
40	VSQGTSPGWAETK	2		X	X	
33	SNYPGYTYR	1	X	X	X	X
35	TASNYPGYTYR	12	X	X	X	X
5	ALSAAELAERTASNYPGYTYR	1	X	X	X	X
4	ALSAAELAER	7	X	X	X	X
2	AALSAAELAER	1	X	X	X	X
1	AAALSAAELAER	2	X	X	X	X
8	GAAALSAAELAER	5	X	X	X	X

39	VLGAAALSAAELAERTASNYPGYTYR	14	X	X	X	X
38	VLGAAALSAAELAER	38	X	X	X	X
3	AIYYGADR	1		X	X	X
7	ERPIDDPTIEVDF	1	X	X	X	X
6	DTAVQPRPWWVK	4	X	X	X	X

Supporting Figures

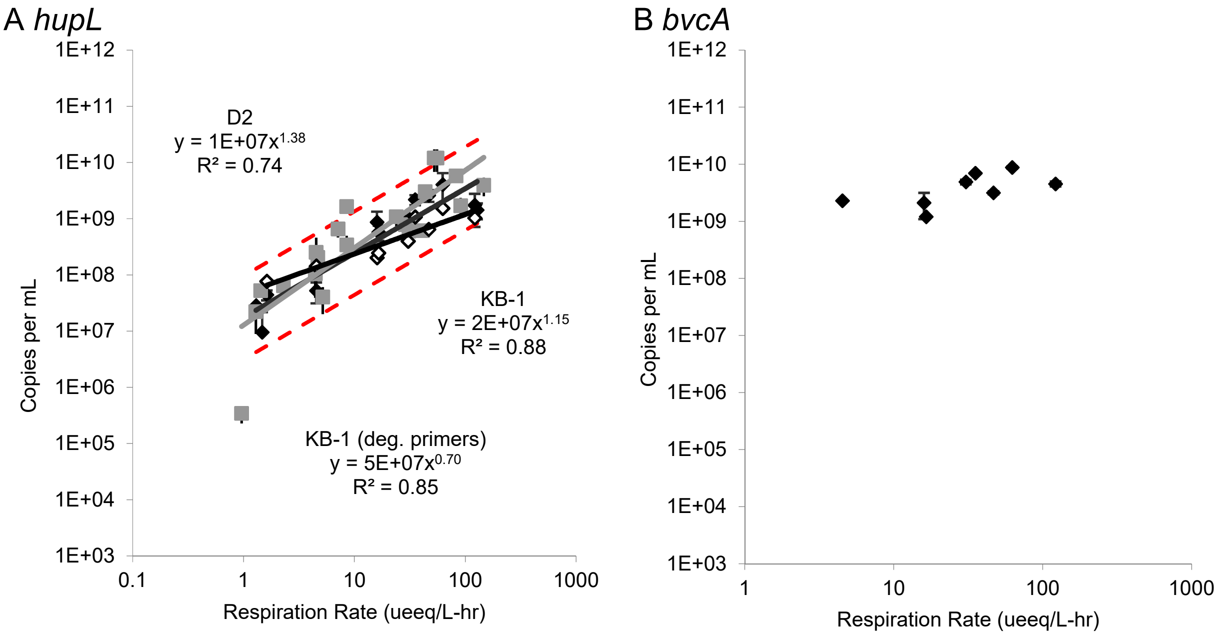

Figure S1: Pseudo-steady-state mRNA concentrations (copies per mL) of specific targets hydrogenase *hupL* (left) and dehalogenase *bvcA* (right) vs. steady-state respiration rates (µeeq/L-hr) for the KB-1 culture (black diamonds) and D2 culture (grey squares). Data for KB-1 with newly designed degenerate *hupL* primers (white diamonds) are also compared against the old primers (black diamonds).

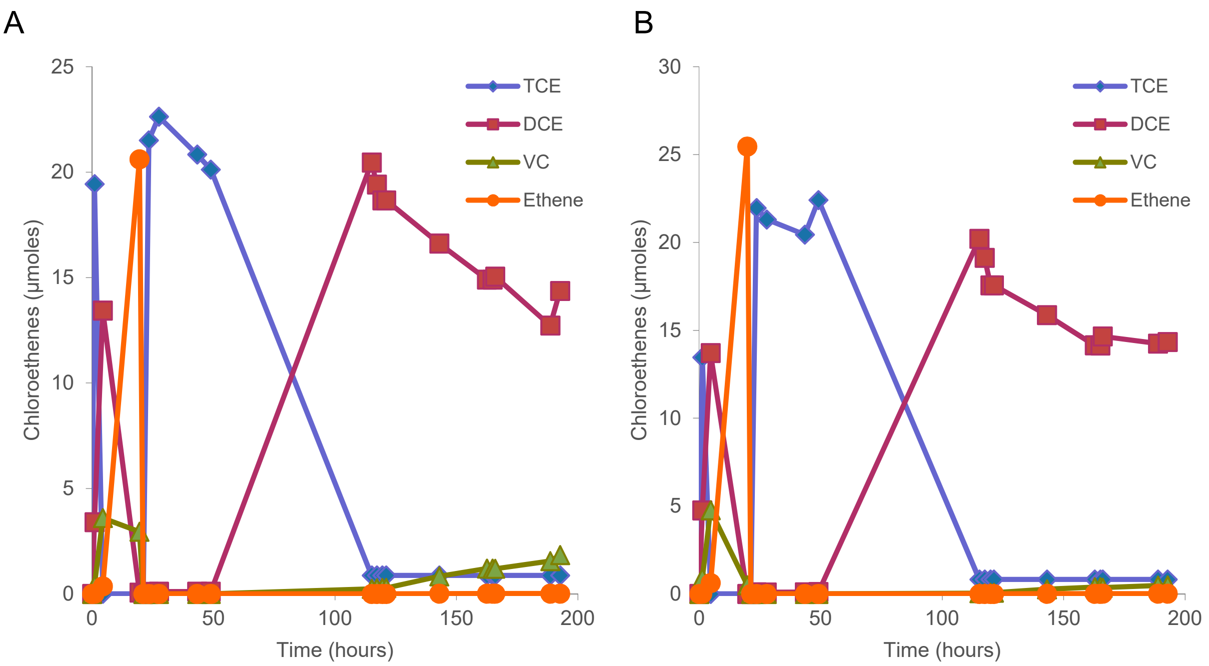

Figure S2. Timecourses of chloroethene data for oxygen-stressed bottles C1 (A) and C2 (B) for the KB-1^{TM} culture. The arrow indicates when the bottles were purged, re-fed TCE, and the oxygen was added.

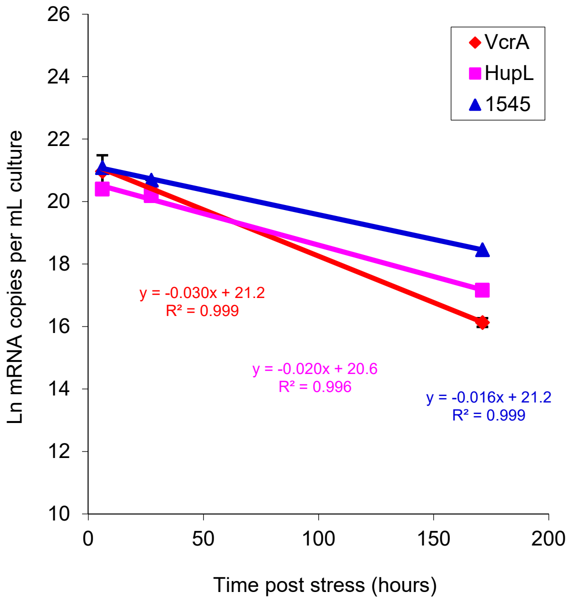

Figure S3. Quantification of mRNA biomarker levels in batch reactors following addition of oxygen (3.14 mg, ~1.6 mg/L aqueous concentration) with exponential decay fits (Ln mRNA vs. time) demonstrating endogenous mRNA degradation. Error bars represent standard error based on biological duplicates.
